# Innovative Patient Involvement During Covid-19: Keeping Patients at the Heart of HTA

**DOI:** 10.3389/fmedt.2021.793119

**Published:** 2021-12-20

**Authors:** Mark Rasburn, Helen Crosbie, Amanda Tonkinson, David Chandler, Tasneem Dhanji, Stephen Habgood, Stella O'Brien

**Affiliations:** ^1^National Institute for Health and Care Excellence (NICE), Public Involvement Programme, Level 1A, Manchester, United Kingdom; ^2^Psoriasis and Psoriatic Arthritis Alliance, 3 Horseshoe Business Park, St. Albans, United Kingdom; ^3^Independent Researcher, Manchester, United Kingdom; ^4^Independent Researcher, Stafford, United Kingdom

**Keywords:** PPI, health technology assessment, patient value, capacity building, new technologies

## Abstract

The COVID-19 pandemic and lockdown measures in the United Kingdom resulted in significant challenges and created opportunities for innovation to keep patients at the heart of HTA. The introduction of the Coronavirus Act 2020 and the associated public health guidance meant that NICE's conventional HTA methods were no longer feasible. NICE introduced rapid, innovative updates to patient and public involvement (PPI), decision-making meetings, and consultations to harness the expertise of patients and the public to ensure guidance addressed the expected concerns and identified barriers which could impact access. This article describes the PPI support for NICE's rapid shift to virtual meetings and virtual engagement. We utilize the authors' experience and patient and public contributor feedback to understand the experience of participating in a virtual setting and identify four themes: accessibility; inclusivity; transparency; and intrapersonal relationships and committee dynamics. The article also considers how patient representatives participated in, and facilitated, the development of guidance for a hypothetical technology to keep patients and the public at the heart of expedited and novel HTA processes to identify and understand the expected patient concerns and potential barriers for when a technology would be introduced.

## Introduction

“*In the early days, watching Covid*−*19 move through the world was like seeing the flood coming. We needed to build an ark around us and get underway at the same time as the waters were rising and the environment changing in unexpected ways while also exposing traditional fault lines of health and socio-economic inequalities.”* NICE patient and public committee member

The National Institute for Health and Care Excellence (NICE) is a world leader in patient and public involvement (PPI) in health technology assessments (HTAs). NICE has pioneered the innovation, iterative development, and evaluation of best practice in all its methods and processes so that the values and standards of meaningful PPI (1) are embedded as a core principle (2).

NICE's PPI framework solicits and incorporates the expertise, experiences and perspectives of lay people, patients and carers, and patient organizations at multiple stages in the HTA process; centers their needs; and acknowledges the outcomes they value most (3).

The exigencies of the COVID-19 pandemic motivated NICE to review and systematically revise its HTA processes to ensure continuity of its mission to support the health care system and provide timely access to effective technologies for patients and the public.

The introduction of the Coronavirus Act 2020 (4) and the associated public health guidance (5) meant that NICE's conventional HTA methods were no longer feasible. NICE introduced rapid, innovative updates to PPI, decision-making meetings, and consultations to harness the expertise of patients and the public to ensure guidance addressed the expected concerns and identified barriers which could impact access. NICE had to continuously evaluate and analyze the impact on PPI and patient contributors and introduced measures to mitigate risk of exclusion and avoid tokenistic involvement. Due to the rapid nature of the updates some of these measures were reactive and implemented at various stages as the organization adapted to the new ways of working.

NICE recognized the potential of new forms of collaboration to disrupt previously identified barriers to PPI such as the resource-intensive need to attend in-person meetings (6). The COVID−19 syndemic (7) also challenged NICE to create a framework to develop guidance for technologies that did not yet exist, and maintain its commitment to PPI, leveraging the expertise of patients and the public to anticipate and address barriers which could impact patient access to such technologies.

The authors of this article are NICE public involvement staff, HTA committee lay members, patient experts and representatives from patient organizations. We describe and reflect on the successes and challenges for keeping patients and the public at the heart of expedited and novel HTA processes by reviewing two innovative approaches; NICE's rapid shift to virtual meetings and virtual engagement, and how we participated in, and facilitated, the development of guidance for a hypothetical technology.

## Innovation One: Introducing Virtual Meetings And Engagement

The first virtual committee meeting NICE held took place on the 24 March 2020 (8). The format of virtual meetings replicated physical meetings; the agenda followed the same structure, the duration of meetings remained the same, and NICE's patient and public involvement principles (9) remained consistent.

Adapting quickly to introduce and support virtual committee meetings and virtual engagement for the first time after more than two decades of physical meetings meant NICE had to learn in real-time what the technology and training requirements were, and the necessary support committee members and stakeholders required to meaningfully engage in this innovative approach.

NICE needed to continuously review individual needs, from ensuring people had the necessary devices and connectivity to enable them to engage, and competence in the use of the virtual engagement software. NICE also needed to understand the differences between virtual meetings and physical meetings that might impact meaningful involvement.

NICE now has 18 months of data capturing the experiences of those involved in virtual committee meetings and virtual engagement to inform the evolution of our processes. The data was generated through exit surveys completed by patient and public contributors to understand their experience of participating in a virtual setting and then thematically analyzed. The data, and the perspective of the authors, has identified notable differences that can be themed into four areas:

1. accessibility
2. inclusivity
3. transparency
4. intrapersonal relationships and committee dynamics.

### Accessibility

From a patient and public perspective, virtual meetings enable greater accessibility and remove barriers that may have prevented or restricted involvement. This is most notable in the removal of the need to travel to physical meetings.

It is recognized that HTAs require evidence from patients with lived experience to reflect on what it is like to live with a condition in real life. Not only does this provide a wider perspective and add to the evidence, but it can also help clarify the circumstances in which different types of evidence have strengths or limitations (10).

Often those with the required lived experience are unable to attend physical meetings, particularly those who have health-related challenges. In addition to attending a meeting, participants would also be required to travel to a physical meeting space; a barrier that restricts the ability to participate in a meeting. Virtual meetings have removed the need to travel, making the opportunity to attend and participate in meetings accessible as it can reduce fatigue and recovery time, which is particularly important to people living with disabilities, long term conditions or side-effects of some treatments.

This has wide-ranging benefits, not just to those with health-related challenges. The removal of travel also removes the geographical barrier that may have prevented participation. This is especially beneficial to those who may live long distances away from the physical meeting space, or those in rural communities and those with limited transport links.

The virtual setting provides an improved opportunity for people to participate, no matter where they are located. This can increase the patient and public population HTA bodies are able to reach, therefore increasing the opportunity to gather a wider range of views and experiences. This increased reach can identify additional needs and outcomes valued most to better reflect patients and the public.

The removal of travel also introduces time-saving benefits. Not only has this been noted to reduce the stress created by the need to travel and ensure arrival on time, but it also enables participants to better manage other commitments and reduces the need to organize additional arrangements. An example of this is those with caring responsibilities or those who need to take leave from work. Virtual committees eliminate the time commitments associated with travel, resulting in a reduction of the total time it takes to participate in a HTA. Participants have reported the removal of travel has enabled them to allocate that time for increased preparation, both by reading the committee materials and getting mentally prepared.

From an administrative perspective, virtual meetings reduce some of the financial costs required for getting people to a physical space, such as the travel bookings, accommodation, and subsistence allowance. Whilst these cost-saving benefits may impact HTA bodies to a higher degree, the removal of financial burdens for participants has a notable benefit to improving accessibility.

Whilst NICE already had a policy to provide travel and accommodation costs upfront (11), additional costs that required up-front payment, such as sustenance allowances, could be a financial barrier to those from lower socio-economic groups. Removing the potential financial burden can aid in removing this barrier to involvement and support equal opportunity.

Virtual meetings do present additional risks of exclusion. One risk is excluding those who have low digital literacy or do not have the financial resources to participate virtually, such as not owning a computer and experiencing data poverty. Another risk is excluding those who do not have a quiet or private space to participate in virtual meetings.

Whilst NICE uses a video teleconference platform that is free for external audiences, participants still require the hardware to enable them to participate and the knowledge to use the software. To mitigate this potential barrier, NICE introduced reasonable adjustments to offer additional support to ensure participants had the resources to be able to attend. This included providing reasonable expenses to ensure there was not an inequality to participation due to communication technology poverty and relatively poor digital infrastructure, such as not having access to a computer or a reliable internet connection. NICE also introduced technical training before meetings to ensure participants can use the software and provides live technical support.

Another risk was excluding parents or carers who might otherwise have had complex care arrangements. As well as the difficulty of engaging parents who needed to home-school, some participants still needed to book a carer to have privacy and be able to have full attention at meetings; something which was not always possible with lockdown restrictions.

Some participants have also highlighted that reading documents on a screen can be difficult, especially for those who are color blind and need documents printed in an appropriate color and contrast. Due to the social distancing measures that meant staff worked remotely, NICE was unable to access printing facilities. Instead, NICE introduced reimbursement in the form of printing allowance to ensure participants who needed this accommodation could claim this back.

### Inclusivity

Virtual meetings are felt to be more accessible and inclusive. This allows for greater representation of input from all, which supports our values and behaviors of inclusivity, equality and diversity that guide our work, and supports our charter that values the input from patients, carers, and the public (12).

Participants have reported a greater sense of comfort when participating in meetings, resulting in a reduced feeling that involvement is daunting. People can participate from home, so they are able to have greater control over the environment, such as using their own furniture, control the temperature, move around freely, and take additional rest breaks.

There have been notable changes in the facilitation of committee meetings. In physical meetings, people with hearing difficulties relied on adjustments, such as seating arrangements and assistive technologies such as hearing loops. In virtual settings, participants have full control in adjusting the settings to enable them to better participate. An example of this is being able to adjust the volume on their computer to improve audibility and hear everyone clearly. The front-facing camera, and ability to see the person speaking on full screen, also enables people who lip-read the ability to better view those who are speaking, as opposed to sitting around a table with various obstructions blocking their line of vision. To achieve the maximum benefit in this area, all participants are required to have appropriate lighting and be fully in the center of the frame when speaking.

There are also benefits in virtual meeting functions, such as the “raised hand” function. This notifies the Chair that participants would like to speak, and places them in a queue in order of who raised their virtual hand first. This ensures Chairs can see when someone wants to speak, which reduces participants needing to try and notify the Chair. It also disrupts the hierarchy of speakers and disproportionately dominant contributors by clearly indicating who raised their hand first to enable a fair order of speakers.

### Transparency

In the same way virtual meetings have improved accessibility for patient and public contributors, a notable benefit is the increased access to committee meetings for stakeholders and external audiences.

Virtual meetings are not as restrictive in space when compared to physical meeting rooms, and the removal of cost and time implications associated with travel allows and encourages more public observers to attend. This increased attendance helps to increase the transparency of how evidence is scrutinized and enables more people to observe the decision-making process.

Virtual committees also provided additional opportunities to support future contributors by enabling them to attend and observe a committee meeting prior to their own engagement. This enables external stakeholders and patient and public contributors to better understand the processes, what to expect in their committee meeting, the committee membership, the types of questions asked, and the committee dynamics.

### Intrapersonal Relationships and Committee Dynamics

Whilst virtual engagement has brought many benefits, we need to identify and understand the new barriers to meaningful PPI virtual spaces introduce, and develop methods to overcome these. One of these barriers is the restricted opportunity to form interpersonal relationships between committee members. This relationship-building through informal conversations, getting to know each other, discussing ideas and sharing notes usually takes place before and after the meeting, and during breaks. In virtual spaces this opportunity to speak outside of the formal setting has been significantly reduced, and so measures to include additional informal engagement opportunities are required. For example, for some decision-making committees NICE invites the Chair and lay members to technical engagement calls and to join a virtual break-out room with clinical and patient experts prior to the meeting.

There are also challenges that a virtual setting can reduce the flow of conversations and opportunities to bounce ideas off each other due to the impersonal setting. There is also a distinction in the inability to read people's body language, facial expressions, and non-verbal communication. This can increase the difficulty in gauging reactions.

## Innovation Two: Developing Guidance For A Hypothetical Technology

Another innovative PPI approach during COVID-19 was the requirement to react to an emergence evidence base in real-time. A case study for this was the development of an exploratory hypothetical economic modeling of severe acute respiratory syndrome coronavirus 2 (SARS-CoV-2) viral detection point of care tests (13). However, as there was no specific technology being discussed at the time an innovative PPI approach was required to ensure the committee could develop a framework to:

- consider the value of a technology that didn't yet exist for SARS–CoV−2 viral detection point of care tests and serology tests;
- discuss a disease for which the knowledge-base was emerging in real time;
- understand the complex systems into which this innovative technology would be introduced and whether aspects might have the potential to cause additional harm to some demographics in ways that couldn't be incorporated into cost-effectiveness models;
- explore the economic modeling of supporting those developments at scale as well as the potential value to individuals and wider groups.

Two patient experts co-produced patient input for the decision-making committee. They were aware that a hypothetical, reliable, appropriate diagnostic testing would have a substantial role in removing some of the burden of implementation and management for patients, informal carers, and their social networks of support. They felt it would be essential for the functioning of society, from education, and civic involvement to the personal and economic security of much of the population. They anticipated that the long-term consequences of some funding, technical, and social decisions might fall disproportionately on some groups that were already disadvantaged.

Due to the hypothetic nature of the topic, patient experts could not draw on personal experience as no specific test was being discussed. Instead, they explored several health and social care scenarios in which tests might be deployed by drawing on their professional experience and personal caring experience. They used this experience to understand the design requirements, accessibility and usability issues, and issues around trust for introducing novel technologies into complex systems, especially in potentially exigent circumstances. This enabled them to propose outcomes relevant to patients and the public, as well as social and other barriers that reflected responses to similar technologies.

Presenting the expected concerns and potential barriers at an early stage increased the committee's understanding of patient and public needs and desired outcomes, enabling discussions to focus on the impact on those requiring the tests.

“*I think that our presentation did make the discussion focus on “real people,” and how the technology and implementation of it might be perceived by service users. It was difficult to assess how well the issues raised were received, or whether they will make an impact going forward, given the unusual circumstances of the discussions which were based on a hypothetical model in a hypothetical hospital setting.”* NICE patient and public committee member

## Discussion

The COVID-19 pandemic and lockdown measures in the United Kingdom resulted in significant challenges and created opportunities for innovation to keep patients at the heart of HTA.

NICE introduced rapid, innovative updates to long-established PPI methodologies and adapted these in real-time to ensure they adhered to NICE's patient and public involvement principles (9). The introduction of virtual engagement resulted in many benefits, but it also introduced additional barriers to meaningful involvement. Whilst measures were identified to mitigate the risk of exclusion from the beginning, such as ensuring all committee members were provided training to use the software, other barriers were identified as they came up. This required NICE to embrace a responsive approach to ensure appropriate support and adjustments were able to be identified and introduced in the evolving practice.

Developing guidance for hypothetical situations also demonstrated the benefit of meaningful PPI. Despite the technology not yet being developed, the experience and expertise of the patient experts ensured the committee identified and understood the expected health and social care scenarios. This ensured committee decisions focused on the impact of those requiring the technology, resulting in a framework that addressed the expected concerns and potential barriers for when a technology would be introduced.

The unprecedented lockdown situation was a significant driver for these changes. The legacy of increased inclusivity, accessibility, transparency, and impact should be commended as a positive in the practice of PPI. An additional legacy should be the realization that HTA bodies have access to people who are familiar with some of these drivers and have the experience of using the technology and understanding of the relevant issues. This can assist establishing best practice from the outset. The culture of reacting quickly to change and embracing novel approaches also needs to be continued and nurtured. By doing so, HTA bodies can continue to strengthen approaches to keeping patients at the heart of HTA.

## Data Availability Statement

The data analyzed in this study is subject to the following licenses/restrictions: The article draws on data submitted to the 2021 HTAi conference through a panel discussion. Requests to access these datasets should be directed to mark.rasburn@nice.org.uk.

## Author Contributions

All authors listed have made a substantial, direct, and intellectual contribution to the work and approved it for publication.

## Conflict of Interest

MR, HC, and AT are employed by NICE. The remaining authors declare that the research was conducted in the absence of any commercial or financial relationships that could be construed as a potential conflict of interest.

## Publisher's Note

All claims expressed in this article are solely those of the authors and do not necessarily represent those of their affiliated organizations, or those of the publisher, the editors and the reviewers. Any product that may be evaluated in this article, or claim that may be made by its manufacturer, is not guaranteed or endorsed by the publisher.
